# Implementation Science and Pediatric Diabetes: A Scoping Review of the State of the Literature and Recommendations for Future Research

**DOI:** 10.1007/s11892-024-01561-3

**Published:** 2024-10-29

**Authors:** Julia Price, Jaclynn Hawkins, Daniel J. Amante, Richard James, Debra Haire-Joshu

**Affiliations:** 1 Center for Healthcare Delivery Science Sidney Kimmel Medical College, Nemours Children’s Health, Thomas Jefferson University, Rockland Center I, 1600 Rockland Road, Wilmington, DE 19803 United States; 2 School of Social Work, University of Michigan, Michigan, United States; 3 Department of Population and Quantitative Health Science, UMass Chan Medical School, Worcester, United States; 4 Medical Library, Nemours Children’s Hospital Delaware, Wilmington, United States; 5 Center for Diabetes Translation Research, Washington University in St. Louis, St. Louis, USA

**Keywords:** Implementation science, Pediatric, Diabetes, Evidence-based care

## Abstract

**Purpose of Review:**

This scoping review aimed to identify implementation science (IS) research in pediatric diabetes, report integration of IS theory and terminology, and offer guidance for future research.

**Recent Findings:**

Of 23 papers identified, 19 were published since 2017 and 21 focused on type 1 diabetes. Most involved medical evidence-based practices (EBPs; *n* = 15), whereas fewer focused on psychosocial (*n* = 7) and diabetes education (*n* = 2). The majority either identified barriers and facilitators of implementing an EBP (*n* = 11) or were implementation trials (*n* = 11). Fewer studies documented gaps in EBP implementation in standard care (*n* = 7) or development of implementation strategies (*n* = 1). Five papers employed IS theories and two aimed to improve equity.

**Summary:**

There is a paucity of IS research in pediatric diabetes care literature. Few papers employed IS theory, used consistent IS terminology, or described IS strategies or outcomes. Guidance for future research to improve IS research in pediatric diabetes is offered.

## Introduction

Decades of research have led to numerous evidence-based practices (EBPs), clinical practice guidelines, and standards of care focused on improving pediatric diabetes care for youth and families [1–7]. These EBPs address multiple aspects of care, including diabetes management, psychosocial needs, diabetes education, diabetes advanced technologies, and school-based care [1–7]. These EBPs lead to improved short and long-term physical and mental health outcomes [1–7]. However, it may take up to 17 years for research findings to be integrated into standard healthcare [8–10]. Research focused on integrating EBPs into the standard pediatric diabetes care families receive across the U.S. and internationally is critical. With documented inequities in access to pediatric diabetes care and in diabetes outcomes, efforts to expand the reach of pediatric EBPs must also include decreasing disparities [11–14]. Implementation science offers rigorous methods as well as models, theories, and frameworks to improve the research-to-practice lag time and address inequities in pediatric diabetes care [15].

As defined by Lane-Fall and colleagues (2019), implementation science is comprised of at least three broad and connected lines of inquiry aimed at closing the research-to-practice gap: (1) understanding the factors that influence the implementation of an EBP, (2) developing strategies to improve the implementation of the EBP, and (3) testing of these strategies [16]. For example, depression screening in youth with type 1 diabetes (T1D) is an EBP [4]. A foundational step in implementation science is to document gaps in the delivery of an EBP as part of standard care (i.e., research-practice gaps). Survey-based studies and electronic medical record review studies may evaluate the percent of eligible youth who are screened for depression or diabetes medical complications with an evidence-based screening tool across one or more clinics.

If the selected EBP is not universally delivered as part of standard pediatric diabetes care, a next step in implementation science research may be to examine implementation barriers and facilitators in the desired context (e.g., outpatient pediatric diabetes clinic). Such studies often involve qualitative or mixed methods to understand perspectives from varied stakeholders (e.g., patients, caregivers, providers, healthcare leaders). Next, researchers may use these data to develop implementation strategies to improve the integration of the EBP, such as depression screening, into standard practice. Implementation strategies are methods to improve the process of integrating an EBP into routine care [17]. Common examples of implementation strategies include conducting educational meetings, developing a formal implementation blueprint, and auditing and providing feedback. Implementation strategies can be tailored to address specific barriers and facilitators using specific, stepwise rigorous methods to select and tailor strategies for each barrier (i.e., implementation mapping, evidence-based quality improvement) [18, 19].

Finally, implementation strategies are tested in implementation trials, which often evaluate clinic-level changes in the delivery of an EBP [20]. In their seminal paper, Proctor and colleagues (2011) offered the gold standard definition of eight implementation outcomes [20]. Examples of implementation outcomes include the adoption of the EBP in standard care (e.g., number of people receiving the EBP compared to those eligible) and the fidelity of delivering the EBP completely and accurately as part of standard practice [20]. Hybrid effectiveness-implementation trials may consider individual changes in health outcomes (e.g., glycemic levels) in addition to implementation outcomes [21]. Implementation trials require large multi-site studies to evaluate implementation outcomes and should compare effectiveness of implementation strategies to meaningfully improve delivery of the EBP.

Models, theories, and frameworks guide each of these areas of implementation science [22]. Determinants frameworks can be used to define multi-level barriers and facilitators and evaluation frameworks can be used to assess impact of implementation strategies [23, 24]. Using such frameworks to guide implementation research is critical to advancing our understanding of how and why implementation of an EBP fails or succeeds. A growing consensus on terminology, guidance on reporting standards (e.g., implementation strategy descriptions), and integration of methods (e.g., mixed qualitative-quantitative methods, community-engaged approaches) enhance reproducibility and rigor in this field [25]. Infusing equity throughout these areas of implementation science is necessary to overcome disparities in care and outcomes [26]. For example, selecting EBPs (e.g., evidence of efficacy and effectiveness in diverse samples) and designing implementation strategies (e.g., include diversity in key informants) should include consideration of how such choices may exacerbate or alleviate disparities.

While implementation science offers an opportunity to effectively integrate EBPs into care, a dearth of implementation science research on pediatric diabetes exists. A review of the pediatric diabetes and implementation science literature is needed to identify gaps in this field and offer recommendations on ways to advance this work. The current scoping review aims to (1) identify pediatric diabetes medical, psychosocial, and educational care EBPs in implementation science research, (2) report on areas of implementation science research in pediatric diabetes (i.e., documenting research-practice gaps, identifying implementation barriers and facilitators, developing implementation strategies, conducting implementation trials), (3) evaluate use of implementation science models/theories/frameworks and terminology, (4) document integration of equity considerations, and (5) offer recommendations to expand implementation science research in pediatric diabetes. Such lines of research can improve the reach of EBPs to optimize physical and mental health and quality of life outcomes for all families of children with diabetes.

## Methods

### Data Sources and Search Strategy

Following the JBI guidelines and PRISMA-ScR checklist for scoping reviews and in collaboration with an expert medical librarian (RJ), a search of PubMed, Scopus, PsychINFO, Emcare, CINAHL, and Cochrane Trials Register databases was conducted on 1/31/2023 for English language articles and repeated in 11/2023. Identified articles were uploaded into Covidence software (Melbourne, Australia). At each review phase (abstract/title, full-text), two reviewers (JP, DA, JH, RJ) independently applied inclusion/exclusion criteria to each paper. Conflicts between reviewer decisions were discussed amongst at least two reviewers and consensus was reached.

### Study Eligibility

Eligible studies (1) focused on pediatric patients (i.e., youth 18 years or younger, studies may also include adult participants in addition to pediatric participants) (2) who have diabetes (e.g., type 1 diabetes, type 2 diabetes) and (3) examined an evidence-based practice (EBP; medical, psychosocial or diabetes education-related practice) and (4) the implementation of this EBP. Studies were determined to be focused on implementation of an EBP if they (a) documented research-practice gaps, (b) examined barriers and/or facilitators of implementing an EBP, (c) developed implementation strategies, or (d) tested implementation strategies in a trial. Protocol papers were eligible. Studies that focused on EBPs for diabetes prevention and those that were not available in English were excluded. Non-empirical papers and review papers were also excluded. With a high likelihood of few papers meeting eligibility criteria and awareness that this is a small but growing literature, a decision was made to include protocol papers to capture the latest methods and topics of empirical work in this area.

### Data Extraction

Two reviewers independently extracted data points from each included manuscript and one reviewer (JP) resolved conflicts, consulting with other reviewers as needed. To ensure consensus on defining the area of implementation research and EBP for each included paper, three primary reviewers met and agreed on these data elements for each paper. Four areas of implementation research included: (1) research-practice gap, (2) barriers and facilitators, (3) implementation strategy development, and (4) implementation trial.

## Results

**Fig. 1:**
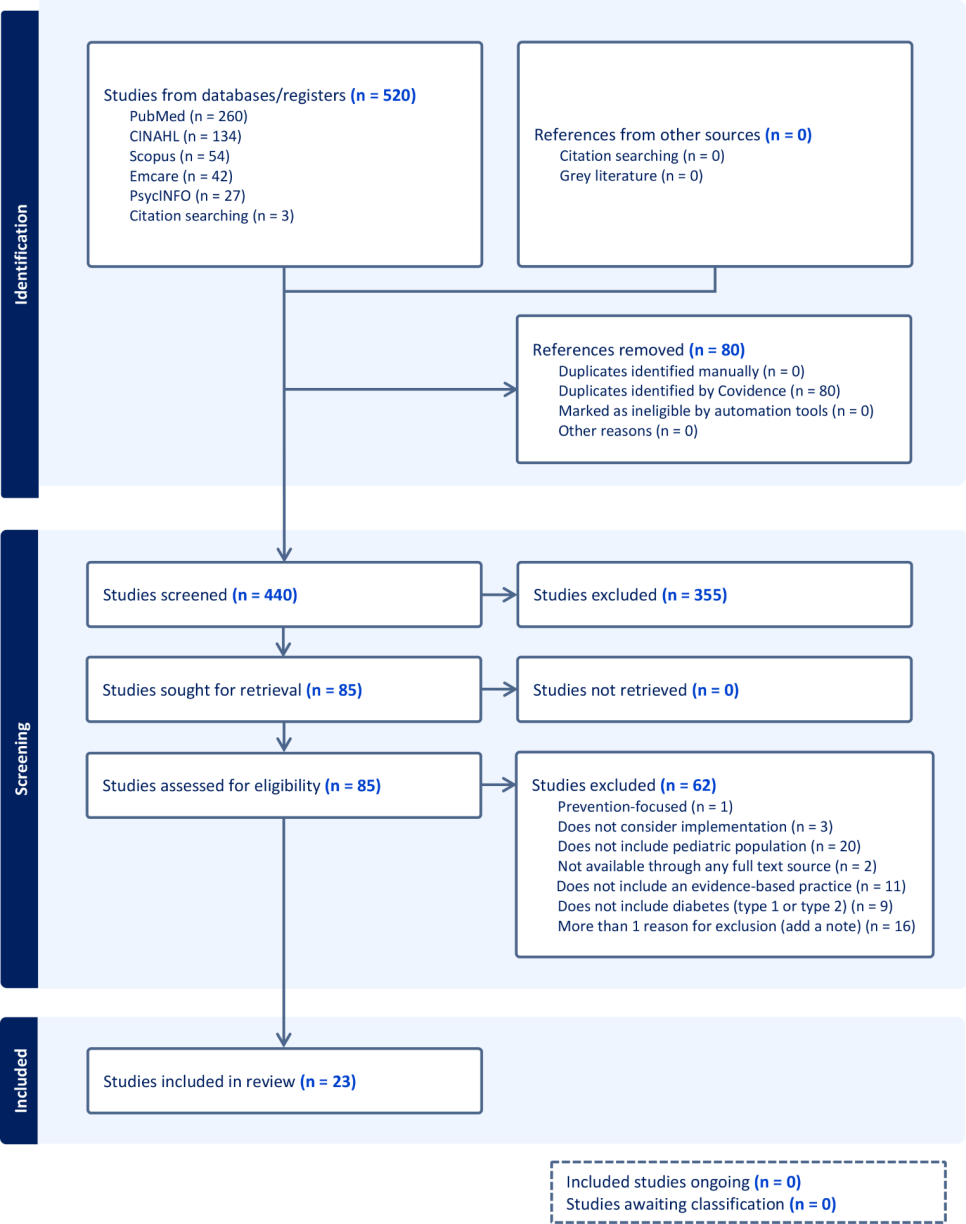
PRISMA flowsheet

Following removal of duplicate papers, 156 studies were identified in the initial search, 62 reached full-text review, and 23 were included in analyses (see Fig. 1). Of the 23 included papers, 19 (82.6%) were published between 2017 and 2023 and 21 (91.3%) focused on T1D. Many of the studies were conducted in the United States (*n* = 10) or the United Kingdom (*n* = 3) or were conducted using the international SWEET registry (*n* = 3). Fifteen studies reported extramural funding, with the most common funding sources being the National Institutes of Health (*n* = 5), the National Institute for Health and Care Research (*n* = 3), and SWEET (*n* = 3).

Most studies involved medical EBPs (*n* = 12; see Table 1), whereas fewer focused on psychosocial (*n* = 7; see Table 2) and diabetes education (*n* = 2; see Table 1) EBPs [27–49]. Two studies considered interventions to support the transition from pediatric to adult diabetes care (see Table 2). Most studies (*n* = 11) identified barriers and facilitators of implementing an EBP or were implementation trials (*n* = 11); fewer studies documented gaps in the use of an EBP in standard clinical practice (*n* = 7). Only one paper detailed the development of an implementation strategy. Five papers employed implementation science theories/models/frameworks. While 13 of the 23 papers mentioned equity, most did so as part of a discussion of their data or next steps for research (*n* = 11), whereas few papers reported aims to improve equitable access to care or health outcomes (*n* = 2).

**Table 1:** Medical care and diabetes education implementation studies in pediatric diabetes

First Author	Year	Type of Diabetes	Evidence-Based Practice	Implementation Focus	Theories/Models/ Frameworks	Equity Considered?
Medical Care						
Margeirsdottir [40]	2010	1	ISPAD Guidelines for medical care	Implementation Trial	Quality Improvement	No
Montgomery [42]	2013	1	Screening for microalbuminuria	Implementation Trial	No	Yes
Zahanova [49]	2017	1	Clinical Practice Guidelines for Screening for diabetes-related complications	Implementation Trial	No	No
Al Nemri [27]	2017	1	Best practices for inpatient management of DKA	Implementation Trial	ADAPTE	No
Manifold [38]	2019	2	Simplified T2D screening with A1c	Barriers & Facilitators; Implementation Trial	No	Yes
March [39]	2020	1	School-based diabetes care, advanced diabetes technology	Barriers & Facilitators	No	No
Cardona-Hernandez [33]	2021	1	Advanced diabetes technology for self-management	Research-Practice Gap	No	Yes
Wolf [48]	2021	1 & 2	Ophthalmic screening guidelines to diagnose diabetic retinopathy	Implementation Trial	No	Yes
Limbert [37]	2022	monogenic	Diabetes-related antibody testing to screen for monogenic diabetes	Research-Practice Gap	No	Yes
An [28]	2022	1	School-based diabetes care	Barriers & Facilitators	No	Yes
Papoutsi [44]	2022	1 & 2	Diabetes medical care (group-based approach)	Implementation Strategy Development; Implementation Trial	Complexity Theory	Yes
Cockcroft [35]	2023	1	NICE Guidelines for physical activity + diabetes management	Research-Practice Gap; Barriers & Facilitators	Capability, Opportunity, and Motivation Model of Beh.	No
Diabetes Education						
Waller [47]	2005	1	Diabetes education, DAFNE adapted for pediatrics	Research-Practice Gap; Barriers & Facilitators	No	No
Nilsson [43]	2018	1	Hospital-based Home Care for new onset diabetes	Barriers & Facilitators	Promoting Action on Res. Implem. in Health Services	No

Note. NICE = National Institute for Health and Care Excellence. DAFNE = Dose Adjustment For Normal Eating

**Table 2:** Implementation papers on psychosocial care for pediatric diabetes and transition to adult care

First Author	Year	Type of Diabetes	Evidence-Based Practice	Implementation Focus	Theories/Models/ Frameworks	Equity Considered?
Psychosocial Care						
de Wit [36]	2014	1	ISPAD Clinical Consensus Guidelines for Psychological Care	Research-Practice Gap; Barriers & Facilitators	No	No
Marker [41]	2019	1	Depression screening; Pediatric Health Questionnaire	Implementation Trial	Quality Improvement	Yes
Brodar [30]	2021	1	Psychosocial screening and diabetes management screening	Implementation Trial	No	Yes
Brodar [31]	2022	1 & 2	Psychosocial screening & care via telehealth during pandemic	Implementation Trial	Quality Improvement	No
Brodar [32]	2022	1	Psychological screening and consultation	Barriers & Facilitators	No	Yes
Chobot [34]	2023	1	ISPAD Clinical Consensus Guidelines for Psychological Care	Research-Practice Gap; Barriers & Facilitators	No	No
Price [45]	2023	1	ISPAD Clinical Consensus Guidelines for Psychological Care	Research-Practice Gap; Barriers & Facilitators	No	Yes
Transition to Adult Care						
Shulman [46]*	2019	1	Transition from pediatric to adult care; Got Transition	Implementation Trial	Quality Improvement	Yes
Bindiganavle [29]	2022	1	Transition Readiness Assessment Questionnaire	Barriers & Facilitators; Implementation Trial	Quality Improvement	Yes

Note. ISPAD = International Society of Pediatric and Adolescent Diabetes

*Protocol paper

### Medical Care for Pediatric Diabetes

#### Evidence-Based Practices

Among medical care studies, there was a relatively even split across screening or assessment (*n* = 6) and treatment (*n* = 7) EBPs. Screening studies included screening for diabetes complications (including microalbuminuria and ophthalmic issues), monogenic diabetes, T2D, and implementation of ISPAD medical care guidelines. Studies examining implementation of EBPs for pediatric diabetes treatment included in-hospital management of diabetes ketoacidosis (DKA), group-based medical care, and guidance on physical activity. Two studies examined school-based diabetes medical care, one of which examined use of advanced diabetes technologies (i.e., insulin pumps, continuous glucose monitors, hybrid-closed loop insulin infusion systems) in schools.

#### Areas of Implementation Research

Only 3 studies documented research-to-practice gaps, two of which employed the Better Control in Pediatric and Adolescent Diabetes: Working to Create Centers of Reference (SWEET) international registry. First, one SWEET registry study examined use of diabetes technology (insulin pumps, continuous glucose monitors) across 101 SWEET centers, showing that > 60% of the 25,654 pediatric T1D patients used at least one technological component for diabetes management [33]. Second, in a survey study of 105 SWEET diabetes centers, 79 clinics reported on access to antibodies testing (84%), C-peptide determination (73%), and genetic testing (57%). In addition, inequities in access to antibody testing across higher and lower income countries were related to clinical outcomes [37]. Third, in an online survey study of 77 pediatric diabetes units in England and Wales (2019–2020), healthcare providers (*N* = 114) reported that their role includes supporting (94%) and promoting (88%) physical activity in their patients, consistent with NICE guidance [35].

Four studies identified barriers and facilitators of implementing an EBP. Results from a survey study in England and Wales (described above) indicated that barriers to implementing an EBP around physical activity guidance largely focused on provider factors, such as lack of time, minimal knowledge about the EBP, and limited training opportunities [35]. Two qualitative school-based studies identified barriers of implementing diabetes care and use of advanced technology in that setting [27, 39]. Both studies included small samples of public school nurses (11 to 40) who either identified as White or served largely White students. Multilevel barriers included parental expectations, inconsistent training on use of technologies, interpersonal communication and cooperation, school organizational factors, and local and school policies. A fourth study employed qualitative interviews with medical staff to identify barriers to screening for T2D in a remote town in the Kimberley region of Australia [38]. Barriers included the complexity of the screening guideline, provider apprehension about screening in young adolescents, and organizational issues (e.g., use of automated recall system to achieve patient follow-up, limited time, high use of locum staff).

Only one study described the development process for an implementation strategy, specifically a group-based delivery method for evidence-based pediatric diabetes care [44]. These authors did not identify this delivery method as an implementation strategy. This mixed methods study involved development and refinement of the group-based medical care model at two implementation sites in the United Kingdom serving 78–80% adolescent and young adult patients who identify as ethnic minorities.

Seven studies involved implementation trials. Nearly half of the implementation trials occurred at single clinics (*n* = 3) and half were multisite trials (*n* = 4) that ranged from 2 to 25 sites (one study not specifying the number of sites). Although no study used the term implementation strategy, three examined locally developed clinical practice guidelines or screening algorithms as strategies to increase use of an EBP [27, 38, 42]. Two studies described testing technology-based implementation strategies, such as a point-of-care decision aid (iSCREEN) and an artificial intelligence-based approach to ophthalmic exams [48, 49]. In one trial aimed at increasing delivery of evidence-based pediatric diabetes care, an implementation strategy of anonymous comparison on quality indicators among a nationwide system of 25 treatments centers was evaluated [40]. While no studies employed Proctor’s implementation outcomes explicitly, six studies described adoption as the primary or sole implementation outcome and one study did not report an implementation outcome. For example, in a prospective study evaluating the use of artificial intelligence to ophthalmic exams at one clinic (310 pediatric patients, 57% White, 32% Black, 47% male) led to a pre-post increase from 49 to 95% in exam completion and adherence to guidelines [48]. Two studies measured both effectiveness and implementation outcomes (i.e., hybrid studies) [40, 48]. None of the trials included a comparison implementation strategy or set of strategies and the descriptions of implementation strategies being tested were minimal. Rather, pre- and post-implementation data were typically compared (*n* = 6).

#### Models/Theories/Frameworks and Terminology

Taken together, most studies did not employ implementation science terminology, reporting standards, or models, theories, and frameworks. No studies employed the terminology “implementation strategies” and only 3 studies used implementation science models/theories/frameworks, namely complexity theory, ADAPTE, and the Capability, Opportunity, and Motivation Model for Behavior (COM-B) [27, 35, 44, 50–52].

#### Consideration of Equity

Of the 12 medical studies, 7 at least noted issues of equity related to the EBP of interest in the discussion of findings or areas for future research. Only 2 studies aimed to improve equity. One of these studies focused on a group-based implementation strategy to increase the reach of evidence-based medical care among young people with diabetes in “ethnically diverse, and socio-economically deprived settings” [44]. The second study involved a trial to improve screening for T2D among underserved Aboriginal and/or Torres Strait Islander youth [38].

### Psychosocial Care for Pediatric Diabetes

#### Evidence-Based Practices

All seven psychosocial EBP studies included screening or assessment EBPs and some also considered consultation, intervention, or treatment (*n* = 4) [30–32, 34, 36, 41, 45]. Three studies examined implementation of the International Society of Pediatric and Adolescent Diabetes (ISPAD) Clinical Consensus Guidelines for Psychological Care [34, 36, 45]. Only three studies reported specific screening tools used. Two independent groups examined implementation of the Patient Health Questionnaire (PHQ-9), an evidence-based screening tool for depression [30, 31, 41]. One group examined the same screening protocol in two papers, which included the General Anxiety Disorder-7 (GAD-7) for anxiety, Diabetes Stress Questionnaire for Youth (DSQY), Diabetes Self-Management Profile self-report (DSMP-SR), Diabetes Family Conflict Scale, and the Brief Multidimensional Student Life Satisfaction Scale -PTPB version [30, 31]. None of the included studies reported on implementation of a specific evidence-based psychosocial treatment or intervention for youth with diabetes or their families.

#### Areas of Implementation Research

Of the 7 studies, 3 documented research-to-practice gaps using provider-reports on surveys [34, 36, 45]. One survey study of 76 (68% response rate) SWEET network clinics found that 89% offer psychological services [34]. Two other survey studies examined implementation of the ISPAD Psychological Care Guidelines among ISPAD listserv members (21.1% response rate, 155 participants) and among pediatric diabetes clinics across the United States (85% response rate; 95 medical and 86 psychosocial providers from 98 clinics) [36, 45]. Results indicate that psychosocial care is often available at pediatric diabetes clinics, particularly larger clinics [36, 45]. However, consistent implementation of ISPAD Psychological Care Guidelines is limited, including delivery of evidence-based screening and intervention (< 55% of clinics in the United States) [45].

Four studies identified barriers and facilitators of implementing a psychosocial EBP [32, 34, 36, 45]. Results from 3 provider-survey studies and 1 qualitative study of providers (*n* = 7) indicate that common barriers to implementation of psychosocial care include funding for psychosocial services and inadequate staffing of qualified mental health professionals [32,34,36,45. For example, in U.S. pediatric diabetes clinics, a psychosocial provider has an average of four hours per week to care for 100 patients [45]. Medical providers identified some challenges to implementing psychological screening and consultations in clinic, such as logistical barriers (e.g., family leaves clinic before seeing psychology after a positive screen), lack of caregiver awareness of problem (e.g., parent doesn’t know child has anxiety), family negative reactions to this care (e.g., stigma of meeting with psychology), and challenges with referring to mental health providers outside of the diabetes clinic (e.g., lack of qualified professionals, family resistance to this care) [32].

All three psychosocial implementation trials focused on increasing screening and consultation [30, 31, 41]. All studies were single site, pre-post trials reporting adoption as the primary outcome. Variable information regarding strategies tested were provided. In a two-year quality improvement initiative across 4 pediatric endocrinology clinics (> 2150 patients with T1D), depression screening (PHQ-2 and PHQ-9) increased from 0 to 75% on average [41]. Strategies used to increase adoption of depression screening included electronic screening forms with automated scoring, automated alerts to providers for positive screens, electronic health record template, weekly data pulls to measure progress, minimal burden on patients (brief screening measure, involvement of family representative in study design), and availability of in-person follow-up with a mental health professional. The two remaining trials occurred at the same single institution. One trial showed increases of in-person psychosocial screening (*N* = 232 adolescents; 83% of eligible patients completed the screening protocol) and referrals to psychology (up 25%), although limited information regarding the strategies tested were noted [30]. The second study was a quality improvement project that occurred at the height of the COVID-19 pandemic and resulted in a return to pre-COVID-19 pandemic levels of screening (up to 56% of eligible patients by study end) and consultation (peaking at 31% in final cycle) [31]. Strategies to increase screening during the pandemic included remote access to psychosocial screeners for families (email to caregivers with link to online screeners), increasing awareness about these screeners among families and medical colleagues (e.g., daily list of patients screened to medical colleagues to start the day, calls to caregivers to confirm receipt of email with link), allowing adequate time for families to complete screeners, and access to technology for remote visits, remote consenting, and warm-hand-offs for psychology consultations.

#### Models/Theories/Frameworks and Terminology

Similar to pediatric diabetes medical care implementation trials, these psychosocial trials largely involved pre-post designs and did not explicitly describe implementation strategies consistent with reporting guidelines, or measure implementation outcomes. Taken together, these 7 psychosocial pediatric diabetes studies were not described as implementation science research and thus did not employ implementation terminology, models, theories, or frameworks, or reporting standards for implementation science studies.

#### Consideration of Equity

Five out of seven studies mentioned equity and offered data regarding differences in access to care and outcomes across youth race, ethnicity, and rurality. For example, one study reported lower rates of depression screening in clinics serving patients with minority race/ethnicity compared to clinics serving predominantly White patients [41]. Differences in access to trained mental health professionals across higher compared to lower income continents and larger compared to smaller clinics were also documented [36]. While no studies aimed to shift care to be more equitable, all three trials were completely or partially conducted in clinics serving racially and ethnically diverse populations.

### Pediatric Diabetes Education

#### Evidence-Based Practices

The two included education studies focused on two models of diabetes education, one as an adaptation to an existing evidence-based curriculum for adults and the other focused on education for families when a child is newly diagnosed with T1D (i.e., Hospital-Based Home Care; HBHC).

#### Areas of Implementation Research

One paper noted gaps in evidence-based diabetes education standard practice. Using surveys of members of the United Kingdom Diabetes register for specialist nurses, 49.5% of specialty nurses (*N* = 96, 74% response rate; 79.2% pediatric diabetes specialty nurses) reported that their program offered formal education, 36.8% reported education about counting carbohydrates, and 32.6% educated about counting portions [47].

Both papers describe single-institution studies aimed at identifying barriers and facilitators of diabetes education. Provider (medical, psychosocial, educators) feedback was the primary method for understanding barriers and facilitators. In the UK specialty nurse survey study described above, barriers to implementing a structured Dose Adjustment for Normal Eating (DAFNE)-type pediatric diabetes education course included concerns for youth understanding complex regimens, provider staffing, and provider time [47]. In a qualitative study guided by the Promoting Action on Research Implementation in Health Services (PARIHS) framework, investigators used ethnographic observations of team meetings at a single diabetes center to identify implicit facilitators and barriers based on the culture of the clinic and providers (*N* = 24; pediatricians, pediatric nurses, dietitians, social workers) [43]. Barriers to implementing an at-home new onset diabetes education programs included resistance to both shifting existing practice (specifically providing structured, age-dependent information) allowing early discharge, as well as provider anxiety for patient safety [43].

#### Models/Theories/Frameworks and Terminology

While one of these two studies employed an implementation science framework, findings are limited by the narrow data collection approaches, limited description of the clinic context, and inclusion of only direct care professionals at one site as research participants.

#### Consideration of Equity

Neither paper examining diabetes education and implementation noted issues around equity.

### Transition from Pediatric to Adult Diabetes Care

#### Evidence-Based Practices

Two studies considered implementation of support tools around the transition from pediatric to adult diabetes care, namely Transition Readiness Assessment Tool (TRAQ) and Got Transition, an evidence-based framework for transition from pediatric to adult care.

#### Areas of Implementation Research

One paper is a protocol for a quasi-experimental pre-post trial with a control group across five health centers in the Ontario Pediatric Diabetes Network (Got Transition) [46]. This trial will evaluate audit and feedback approaches, or strategies, (which compare results to standards or peer performance and providing feedback to providers) for implementing Got Transition to support transition to adult care among 3 cohorts (*n* = 225 each) of young adults with T1D.

The second study involves a single institution quality improvement project to identify barriers of implementing TRAQ as well as to conduct a pre-post intervention evaluation to increase use of this tool (adoption implementation outcome) [29]. Based on discussions with clinic staff (no sample characteristics noted), barriers included lack of time to discuss and document TRAQ and lack of qualified staff to assist with diabetes technology downloads. Although the authors did not use this terminology, the implementation strategies tested in the pre-post trial included additional training for staff and improvements around accessibility and central location of needed materials. Although there was a significant decline in completion of the TRAQ tool from pre- to post-implementation (*N* = 130; 74.5–53.6%; reportedly due to patients receiving care in a satellite site not staffed for TRAQ), there was a significant increase in patients with documented transition goals (0 to 94.6%) and completed transition goals (0 to 83.8%).

#### Models/Theories/Frameworks and Terminology

Only 1 out of 2 transition studies was conceptualized within implementation science, using a framework to guide the project development, including multiple sites, reporting on implementation strategies, and commenting on implementation outcomes.

#### Consideration of Equity

The authors who evaluated implementation of TRAQ discuss the need to prioritize transition planning among populations experiencing social risk factors, such as families with Medicaid. While the trial protocol outlines plans for measuring socioeconomic status, rurality, and race-ethnicity, the authors did not specifically describe aims around equity. Taken together, neither study on transition aimed to decrease disparities in access to transition care or outcomes.

## Conclusions

This scoping review identified a young and growing literature examining implementation of EBPs in pediatric diabetes care, particularly in the medical care of youth with T1D. However, few studies were conceptualized within the field of implementation science and thus do not conform to reporting standards, utilize implementation terminology, or employ model/theories/frameworks from the field. While many papers commented on issues related to equity in access to care, few studies aimed to change care to become more equitable. Such varied approaches to describing implementation and quality improvement trials create a lack of clarity defining the EBP of interest, the implementation strategies being developed and tested, or the implementation outcomes being measured. Even more, no studies considered sustainability. Rigorous research at the intersection of pediatric diabetes and implementation science is poised to improve the lives of all youth and families living with diabetes, including those who experience disparities in access to care and health outcomes.

### Recommendations for Future Research

To advance implementation science in pediatric diabetes requires multilevel initiatives from investigators (e.g., engage in trainings, incorporate implementation science theory) to funders (e.g., setting research priorities). Beyond pediatric diabetes, other fields, including pediatric critical care, pediatric asthma, and adult diabetes, have had similar calls for increases implementation science research and offer relevant guidance on steps forward [53–58]. Practical tips and resources to incorporate implementation science into pediatric diabetes research are provided. Careful selection of references in the section below offers primer level introduction to the field of implementation science, as well as seminal papers.

#### Obtain Training and Consultation

To support development of expertise in implementation science, many resources for reading, training (e.g., Washington University at St. Louis, University of Colorado, University of Pennsylvania Implementation Science Institute) and online tools (e.g., Theories, Models, & Frameworks| Implementation Science at UW (impsciuw.org)) exist [16, 17, 20–26]. For those focused on expertise in other fields, collaborating with implementation science experts on the development, execution, and dissemination of projects may be the preferred path.

#### Consider Implementation Early

There is a growing call for incorporating implementation considerations earlier in the research pipeline, including in the development of EBPs and in efficacy and effectiveness trials [69]. Such calls aim to speed up the research-to-practice pipeline by narrowing the gap in the translational research spectrum [69]. These calls make the question about when a practice is evidence-based less relevant. Regardless of the amount of efficacy and effectiveness data for any given practice, implementation should and can be considered. Curran and colleagues (2022) offer a tool to guide decision making around type of hybrid effectiveness-implementation study or trial to consider based on a number of factors, including strength of effectiveness data for the practice and anticipated need for adaptations [21].

#### Root Research within Implementation Science Models/Theories/Frameworks

Using implementation science models, theories, and frameworks to guide the design, execution, and dissemination of pediatric diabetes research, will afford greater understanding of the mechanisms implementation and facilitate refinement of implementation theories [22]. Researchers may consider employing commonly used and gold standard models/theories/frameworks, such as Consolidated Framework for Implementation Research (CFIR) to examine barriers and facilitators of implementation, ERIC taxonomy, implementation mapping, or evidence-based quality improvement to develop implementation strategies, Proctor’s implementation outcomes and Curran’s definition of hybrid effectiveness-implementation studies to guide implementation trials, Framework for Reporting Adaptations and Modifications-Enhanced (FRAME) for adaptations of EBPs, and Shelton’s Reach Effectiveness, Adoption, Implementation, and Maintenance (RE-AIM) extension or the Transcreation Framework for designing, delivering, and sustaining behavioral interventions in communities to reduce health disparities [17–21, 23, 24, 62, 67].

#### Incorporate Equity throughout

Implementation science leaders have called for a focus on equity, including designing and selecting EBPs specifically for vulnerable populations and low resource communities, developing and testing implementation strategies that aim to reduce inequities in care, adapting EBPs, and measuring outcomes through an equity lens [26]. Community-engaged approaches to implementation studies can facilitate efforts to improve equity [56–59]. Indeed, the National Institute on Diabetes and Digestive and Kidney diseases (NIDDK) has called for advancing stakeholder engagement, such as including patients as true partners in research [60]. The Transcreation Framework and FRAME can guide this work, including examining the relevance, acceptability, and accessibility of existing EBPs that may have been evaluated in randomized clinical trials that did not include diverse samples and adapting such EBPs [62, 67].

#### Identify Collaborators for Large Trials

Large implementation trials require collaboration across multiple institutions and involve experts in various methodologies and content areas. Such studies afford greater power in evaluating implementation outcomes and in conducting hybrid effectiveness-implementation studies. Existing pediatric diabetes research networks (e.g., SWEET, T1D Exchange) offer opportunities for such work.

#### Adhere to Reporting Standards

Consistent use of common terminology and reporting standards will facilitate future reviews and meta-analyses of implementation findings in pediatric diabetes [64]. Investigators should use reporting standards in manuscripts on implementation studies, to describe EBPs, and to report on implementation strategies [25, 65, 66]. Seminal papers offer lists of conceptually unique implementation strategies and outcomes, with clear definitions of each, as well as guidance in tracking adaptations to EBPs and implementation strategies [17, 20, 67, 68]. Consistent use of such reporting standards, as well as an ‘implementation science’ subject heading in manuscripts, will allow for refined comparisons across studies.

#### Set a Research Agenda

Consensus on prioritizing areas of implementation science research and on rigorous methods is needed in pediatric diabetes. The 2021 National Institute for Diabetes and Digestive and Kidney Diseases (NIDDK)’s strategic research plan called for dissemination and implementation science studies to improve the health of all people more rapidly and more effectively [63]. Lessons from other fields with small and growing implementation science literatures offer further guidance on setting research priorities. Using a modified Delphi method to solicit and rank research priorities in maternal health implementation science, researchers developed a list of research areas with the greatest potential to improve maternal healthcare [70]. Results included prioritization of EBPs for implementation, identification of barriers and facilitators likely to have the greatest influence on care, and enumeration of the most important implementation strategies to test for effectiveness. Key implementation science methods to maintain rigor were also put forward, including selecting and adapting implementation strategies to promote equity, best practices for engaging patients and communities, and developing relevant implementation outcomes. The process of gathering leaders in the fields to generate these recommendations and priorities can readily be applied to the field of pediatric diabetes implementation science to increase future impact of the work.

## Data Availability

No datasets were generated or analysed during the current study.
